# Untreated Pain, Narcotics Regulation, and Global Health Ideologies

**DOI:** 10.1371/journal.pmed.1001411

**Published:** 2013-04-02

**Authors:** Nicholas B. King, Veronique Fraser

**Affiliations:** 1 Biomedical Ethics Unit, McGill University, Montreal, Canada; 2 Department of Social Studies of Medicine, McGill University, Montreal, Canada; 3 Department of Epidemiology, Biostatistics, and Occupational Health, McGill University, Montreal, Canada

## Abstract

Pain management is marginalized or ignored, with millions of people worldwide unnecessarily living with untreated pain. Reducing global inequalities in untreated pain requires a concerted global effort, say Veronique Fraser and colleagues, which must attend to the complexity of pain and promote multimodal, multidisciplinary pain management.

**Summary Points**

- Millions worldwide unnecessarily suffer from untreated pain. This burden is highest in the developing world, and among the poor, elderly, mentally ill, children, women, and racial/ethnic minorities.
- Both the biomedical and public health approaches to global health marginalize or ignore pain management, viewing it as a drain on resources that would be better spent on cure or prevention.
- Reducing global inequalities in untreated pain will require a concerted effort by global health funders, institutions, and organizations to place untreated pain at the top of the list of global health priorities. This effort must attend to the complexity of pain and promote multimodal, multidisciplinary pain management from the outset.

Consider these three fictional cases:

Raj is a 32-year-old taxi driver in Delhi, India. During a motor vehicle collision, he sustains a right sided pneumothorax and an open fracture of the right femur. He is admitted to the trauma ward of a government-run hospital.

Maria is a 55-year-old undocumented housekeeper in Los Angeles, California. She has been diagnosed with lung cancer, with metastases to liver and bone. She is being treated at an urban tertiary teaching hospital.

Evangelista is a 46-year-old mother of five in a rural village in Tanzania. She is HIV positive and was recently diagnosed with tuberculosis (TB). She receives directly observed therapy with follow-up at a non-governmental organization (NGO) clinic.

While apparently unrelated, these cases share two important similarities. First, all three represent pressing global health problems. Road traffic collisions, lung cancer, and HIV/AIDS are among the ten leading causes of mortality worldwide [1] and have each been identified as global health priorities [2]–[4]. Second, Raj, Maria, and Evangelista are likely to experience pain that is severe, debilitating, and untreated. Conservative estimates by the World Health Organization (WHO) suggest that 1 million terminal HIV/AIDS patients, 5.5 million cancer patients, and 800,000 trauma patients have little or no access to treatment for moderate to severe pain [5].

There is a growing consensus that freedom from unnecessary pain is a fundamental human right, and the prevalence of untreated pain is a global health catastrophe [6]–[10]. Recent calls to action [11],[12], including one in *PLOS Medicine* earlier this year [13], identify international narcotics regulation as a fundamental cause of the global epidemic of untreated pain. While narcotics regulation reform at the international and national levels is imperative, we suggest that the problem of untreated pain is more complex and requires more comprehensive solutions.

## Untreated Pain: A Global Health Problem

Pain is the most common reason people seek medical care [7]. Acute pain commonly occurs post-operatively, following trauma, or secondary to severe illness; more than 50% of patients report severe to intolerable pain secondary to injury or post-surgery [9]. Surgery and trauma also account for 25% of the burden of chronic pain [14]. Chronic pain currently affects one in five adults, is more prevalent among women and the elderly, and is associated with physically demanding work and lower education [14].

Chronic-malignant pain, which accompanies terminal illnesses such as cancer, HIV/AIDS, multiple sclerosis, end stage organ failure, and chronic obstructive pulmonary disease [15], is a significant cause of disability [10]. As many as 60%–90% of patients with advanced cancer or in the terminal phases of AIDS experience moderate to severe pain [6], and pain is the second most common complaint of the more than 33 million individuals living with HIV/AIDS in the developing world [9]. Chronic-non-cancer pain (CNCP), which includes neuropathic, musculoskeletal, and visceral pain, accounts for 70% of pain experienced by older patients [15].

Untreated pain has a profound impact on quality of life and can have physical, psychological, social, and economic consequences. Inappropriately managed acute pain can result in immunological and neural changes, which can progress to chronic pain if untreated [16]. Clinical outcomes of untreated postoperative pain include increased risk of atelectasis, respiratory infection, myocardial ischemia, infarct or cardiac failure, and thromboembolic disease [16]. Common sequelae of untreated chronic pain include decreased mobility, impaired immunity, decreased concentration, anorexia, and sleep disturbances [9],[10].

Patients with chronic pain often experience social isolation, dependence on care givers, and impaired relationships with friends and family [14], and are four times more likely to experience depression or anxiety than those without pain [10]. The financial burdens of untreated chronic pain—absenteeism, income loss, healthcare costs, and workers compensation—place the same strain on countries as cancer and cardiovascular disease [9]. In the United States, the annual cost of untreated pain is reported to be between US$560–US$635 billion [17]. It has been argued that many of the conditions causing the greatest global disease burdens, such as depression and trauma secondary to motor vehicle collisions or falls, do so through acute or chronic pain [14]. The prevalence of untreated pain is likely to increase as the population ages in many developed nations, and with the increasing global burden of chronic disease and HIV/AIDS [12].

Global inequalities will also likely widen. More than 90% of HIV patients are located in developing countries, and the WHO estimates that 70% of the 20 million new cancer cases predicted by 2020 will occur in the developing world [12]. The need for pain treatment in these countries is exacerbated by their relative lack of access to curative or palliative care, such as surgery and anti-retroviral drugs [12]. Even in countries with advanced health care systems, pain is often under-diagnosed and undertreated [9], particularly among the poor, elderly, mentally ill, children, women, and racial/ethnic minorities [18],[19]. There is also a lack of training for pain management among vulnerable populations, including children and the elderly, leading to increased risk of untreated pain [15]. For these reasons, the Institute of Medicine recently called for a “cultural transformation” in the approach to pain management in the US [17].

Pain management is complex, and certain types of pain, including CNCP, may require multimodal approaches that combine pharmaceutical and non-pharmacological therapies [15]. Nevertheless, current pharmaceutical interventions are generally effective and, in high-income countries, a cheap and readily available means of reducing acute and chronic malignant pain. Opioids are effective in treating moderate to severe pain and have been included on the WHO Essential Medicine list since 1986 [5]. Yet inequalities in opioid availability are widespread and well-documented. Morphine and other strong opioids are unavailable in over 150 countries [15]. Even when they are available, opioids often cost more in low- and middle-income countries [10],[12]. By one estimate, 600 million people alive today will experience negative health impacts due to untreated pain [12].

## The Role of Narcotics Regulation

Access to pain management is a widely recognized human right, enshrined in international law. There should be no serious technical or financial obstacles to global distribution of effective pain treatments. Why then is there so much untreated pain?

Many blame international narcotics regulation, rooted in the 1961 United Nations Single Convention on Narcotic Drugs, which established the International Narcotics Control Board (INCB) to regulate illicit narcotics (e.g., opium, heroin, cocaine) and drugs for scientific and medical purposes (e.g., morphine, codeine). While charged with simultaneously controlling illicit and scheduled drugs, and ensuring the availability of medications, the INCB and other international organizations including the UN Commission on Narcotic Drugs (CND) and the United Nations Office on Drugs and Crime (UNODC) have emphasized prohibition and law enforcement at the expense of access [12].

The regulatory and scheduling requirements imposed by these organizations are complex, presenting special obstacles to low- and middle-income countries, which often ban scheduled medications outright. Other barriers to prescribing or obtaining opioids in these countries include: ineffective drug distribution systems; lack of clear pain management policies; inadequate training of health care workers in pain management; limiting formulations such as oral morphine; limiting prescribing rights to specialties such as oncology and palliative care; and restrictive licensing for health care workers and pharmacies [10],[11]. Many of these regulations are stricter than those mandated by the Single Convention [10],[20]. Moreover, among many vulnerable populations there are strong cultural barriers to accepting pain medication, including fear of addiction and mistrust of medical intervention [12],[21],[22].

Critics contend that international drug control reform is essential for reducing the global burden of untreated pain. Noting that the INCB is “in the conflicted position of both promoting and throttling the drugs it regulates,” Nickerson and Attan recommend transferring its mandate for promoting medical use of licit narcotic and psychotropic drugs to the WHO [13]. Similarly, Taylor recommends that the INCB use the Single Convention's reporting, inspection, and non-compliance procedures to compel countries to expand medical availability of pain medications [12].

## Pain and the Ideologies of Global Health

We agree that reform of international narcotics regulations is a critical step toward reducing global inequalities in untreated pain. However, these inequalities result not only from the overemphasis on prohibition, but also from lacunae in the prevailing ideologies of global health. Anderson notes that global health organizations are “curiously absent” [11] from the effort to address untreated pain. This absence is not anomalous.

Many global health efforts embrace a “biomedical” model, which prioritizes the treatment and eradication of disease. While focusing on cure does not necessarily preclude care, it often means that resources are directed at treating disease, rather than addressing the suffering that it produces [23]. For example, The Bill and Melinda Gates Foundation's Grand Challenges in Global Health include vaccine development and improvement, vector control, improved nutrition, treatment of latent and chronic infections, and improved health measurement in developing countries, but make no mention of pain. Their 2010 Global Health Program Overview contains no reference to pain or pain management [24]. Similarly, the Global Partnership to Stop TB, Roll Back Malaria, and The Global Fund to fight against AIDS, TB and Malaria, all emphasize case detection, treatment, and cure. These efforts, while important, leave little space for managing the pain incurred by patients living with disease. Indeed, the need for pain management is frequently seen as a mark of failure within the biomedical model [23].

Many global health organizations embrace an alternative, “public health” model, emphasizing prevention through modification of human behavior and the physical and social environment. The annual Health Reports issued by the WHO illustrate the public health approach. While the 1997 report *Conquering Suffering and Enriching Humanity* includes the alleviation of pain and reduction of suffering as a priority area [25], it emphasizes prevention and health promotion strategies, and only briefly addresses the subject of pain management within the context of palliative cancer care. The 2004 report *Changing History*, which proposes a comprehensive strategy for addressing HIV/AIDS, makes no reference to treating pain, though it is a chief complaint among terminal AIDS patients [26]. Despite its commendable emphasis on prevention, health promotion, and the determinants of health, the public health model is no more likely to address untreated pain—which, as in the biomedical model, is often seen as a mark of failure.

## Opioids

Focusing too closely on narcotics regulation also risks overemphasizing the importance of opioids. There is increasing recognition that treating *total pain*—the physical, psychosocial, and emotional dimensions of pain—requires a multimodal approach. In addition to opioids, patients may require multimodal pharmacological management, surgery, rehabilitation, physiotherapy, and other interventions. Pain in HIV/AIDS patients is diverse in nature and etiology. Neuropathic pain and pain of a somatic/visceral nature may be directly related to HIV infection, sequelae to AIDS therapies, or unrelated to either. While the WHO analgesic ladder is recommended for treatment of patients with HIV/AIDS, it is not appropriate for all types of pain, which require a multimodal approach [15]. Certain types of pain, such as neuropathic and bone pain, typically do not respond to opioid analgesics, and the efficacy of opioids for treating CNCP remains undetermined [27]. Non-pharmacological approaches to pain management must also be considered when there is no improvement in the pain state or when side effects arise due to medications [15]. While opioids remain a gold standard for acute pain, multimodal approaches to analgesia are now recognized as best practice in postoperative pain management [16].

Increased access to opioids also carries risks if unaccompanied by adequate training and monitoring. North America has witnessed a dramatic increase in prescription opioid-related mortality due to a variety of factors including increased prescription of opioids for pain relief [28],[29], opioid diversion [30], lack of training among non-specialist physicians in pain management [31], and uneven prescription drug monitoring programs [32]. The rise in opioid-related mortality has also been attributed to a general change in pain management philosophy, as experts drew attention to the perceived under-treatment of CNCP and called for greater access to opioid analgesia [29],[30],[33]. This trend offers a cautionary example that opening the global floodgates to opioids without simultaneously addressing pain as a complex phenomenon requiring multimodal approaches, and putting in place adequate training of health care professionals and anti-diversion mechanisms, may do as much harm as good.

## Untreated Pain: The Global Health Priority

Let us briefly return to our fictional cases. The biomedical approach might respond to Raj, Maria, and Evangelista's cases by developing more effective therapeutics and prophylaxis for HIV, TB, and cancer, and better acute care for trauma patients. The public health approach might prioritize prevention of HIV, TB, cancer and motor vehicle collisions through behavior modification, elimination of environmental carcinogens, and construction of safer roads. Yet even if these interventions were implemented today, they would not reduce the pain experienced by Raj, Maria, and Evangelista tomorrow. Moreover, no matter how effective preventive and curative efforts might be, trauma and disease will still occur, and patients will continue to suffer.

Calls to action on untreated pain are numerous, but they will continue to fall on deaf ears as long as the existing players in global health accept the marginalization of pain management in the dominant ideologies outlined above. Moreover, pain management often competes for resources with other global health priorities. These include diseases with high-profile, well-organized advocacy organizations who, consonant with the biomedical model, prioritize searching for a cure. Conversely, in resource-poor settings, pain management for the terminally or chronically ill may be a lower priority than prevention of illnesses that strike the young and healthy [21]. Indeed, some argue that prioritizing pain management diverts attention and resources from cure and prevention, which if successful will ultimately relieve pain as a by-product [22]. Pain is thus seen not only as a failure, but also a drain on resources that would be better spent on cure or prevention.

This need not be so. Funding for pain management, prevention, and cure should not have to compete with one another. While reform of narcotics regulation is vital, it must be accompanied by a concerted effort among global health funders, institutions, and organizations to place untreated pain at the top of the list of global health priorities. This effort must attend to the complexity of pain and promote multimodal, multidisciplinary pain management from the outset. Without this multilateral and comprehensive commitment, patients like Raj, Maria, and Evangelista will continue to suffer unnecessarily.

## Abbreviations

CNCP: chronic-non-cancer pain
INCB: International Narcotics Control Board
TB: tuberculosis
WHO: World Health Organization
